# Clinical relevance of pre‐ and coseasonal sublingual immunotherapy with a 300 index of reactivity 5‐grass SLIT tablet in allergic rhinoconjunctivitis

**DOI:** 10.1002/clt2.12321

**Published:** 2023-12-18

**Authors:** Oliver Pfaar, Ulrich Wahn, Giorgio Walter Canonica, Farah Bahbah, Philippe Devillier

**Affiliations:** ^1^ Department of Otorhinolaryngology, Head and Neck Surgery Section of Rhinology and Allergy University Hospital Marburg Philipps‐Universität Marburg Marburg Germany; ^2^ Charité Universitätsmedizin Berlin Berlin Germany; ^3^ Department of Biomedical Sciences Humanitas University Pieve Emanuele Milan Italy; ^4^ Personalized Medicine, Asthma and Allergy Humanitas Clinical and Research Center IRCCS Rozzano Italy; ^5^ Paris France; ^6^ VIM Suresnes – UMR_0892 & Exhalomics Clinical Research Unit Airway Diseases Department Foch Hospital University Versailles Saint‐Quentin Suresnes France

**Keywords:** allergen immunotherapy, allergic rhinoconjunctivitis, clinical relevance, combined symptom and medication score, grass pollen tablet

## Abstract

**Background:**

There is considerable interest in improving the scoring methods for evaluating the efficacy of allergen immunotherapy (AIT) and to show if this is associated with clinically meaningful results from the patient's perspective. We aimed to assess the efficacy and clinical relevance of a 300 index of reactivity (IR) 5‐grass pollen sublingual immunotherapy (SLIT) tablet in children, adolescents and adults with moderate to severe grass‐induced allergic rhinoconjunctivitis (ARC) with or without controlled asthma using the combined symptom and medication score CSMS_0‐36_.

**Methods:**

The data of the European population that participated in 3 Phase III, international, randomized double‐blind placebo‐controlled clinical trials were analyzed post hoc.

**Results:**

A total of 864 patients randomized to 300 IR 5‐grass tablet or placebo were analyzed. Over the primary evaluation period, the difference in CSMS_0‐36_ between the 300 IR and placebo groups was statistically significant (point estimates: −2.51, CI_95%_ [−3.88; −1.14], *p* < 0.0001 in clinical trial1; −2.31, CI_95%_ [−3.39; −1.23], *p* < 0.0001 in CT2; and −2.31, CI_95%_ [−3.58; −1.03], *p* = 0.0004 in CT3). The relative differences between the 300 IR 5‐grass tablet and placebo were −29.7%, −33.8%, and −26.3%, respectively. The results based on CSMS_0‐36_ were consistent with those obtained with the primary endpoints of the trials and support the consideration of the 2‐point threshold of the CSMS_0‐36_ for clinical relevance of AIT.

**Conclusion:**

Post hoc analysis of 3 CTs with the 300 IR 5‐grass SLIT tablet confirmed its significant and clinically relevant effect in the European population with grass pollen‐induced ARC with or without controlled asthma.

## 1 INTRODUCTION

One of the commonest symptomatic chronic diseases worldwide with increasing prevalence, allergic rhinoconjunctivitis (ARC) is induced by the IgE‐mediated inflammatory response in sensitized individuals after allergen exposure^1^ and results in a chronic, mostly eosinophilic, inflammation of the nasal mucosa and conjunctiva.^2^ Despite the recommended symptomatic treatment, about one fifth to one third of these patients still suffer from uncontrolled nasal and/or ocular symptoms, with a high impairment on quality of life, decreasing work productivity, social interactions, and other aspects of life.^3^, ^4^ Allergen immunotherapy (AIT) as sublingual or subcutaneous administration is a disease modifying treatment for patients with troublesome disease^5^, ^6^ showing the same clinical efficacy; the patient may choose the one or the other.^5^, ^7^, ^8^, ^9^, ^10^

The 300 index of reactivity (IR) 5‐grass pollen sublingual immunotherapy (SLIT) tablet has been shown to be effective in controlling the symptoms and reducing the need for symptomatic medication in patients with grass pollen‐induced ARC with or without controlled asthma.^11^, ^12^, ^13^, ^14^ Here we present the post hoc analysis in the European population of three randomized, double‐blind placebo‐controlled trials (DBPCT) with the 300 IR 5‐grass tablet in different age groups (children, adolescents and adults).

We aimed to confirm the efficacy of the 300 IR daily dose of this SLIT tablet in grass pollen ARC using a standardized and globally harmonized method for analyzing the clinical efficacy of AIT products in randomized controlled trials. The recommended method by a Task Force of the European Academy of Allergy and Clinical Immunology (EAACI) Immunotherapy Interest Group for optimal endpoints in AIT trials for ARC, in line with both the US Food and Drug Administration (FDA) and the European Medicines Agency (EMA) guidelines, uses a homogeneous combined symptom and medication score (CSMS, scale 0–6) as the primary outcome.^15^ The score considers symptoms as well as medication in a balanced relation and enables a comparison of the daily burden of the disease between different clinical trials.^15^ In addition, we examined how the treatment effect versus placebo translates into a clinically relevant improvement perceivable by the patients.

## 2 MATERIAL AND METHODS

### 2.1 Study design and patients

The design and main results of the DBPCTs have been published before.^11^, ^12^, ^13^, ^14^

Briefly, the efficacy of the 300 IR 5‐grass SLIT tablet administered around 4 months prior to the pollen season and continued for its duration was evaluated versus placebo in 3 randomized DBPCTs (ClinicalTrials.gov numbers: NCT00367640 (CT1), NCT00418379 (CT2) and NCT00409409 (CT3)). Participants with moderate‐to‐severe grass pollen‐induced ARC with or without controlled asthma were aged 18–45 years in CT1 and CT2, whereas children and adolescents aged 5–17 years were included in CT3. Patients were treated pre‐coseasonally over a single year in CT1 and CT3 or discontinuously over 3 consecutive years in CT2. In this trial, the primary evaluation period was at Year 3 and patients were followed‐up during 2 subsequent treatment‐free years.

The three DBPCTs were conducted in Europe. CT2 was also carried out in Canada and Russia, where participants accounted for less than 15% of the overall study population. In this post hoc analysis, we focussed on the European population to ensure data consistency as it has been acknowledged that differences in patients' clinical characteristics and variations in allergen exposure across regions might interfere with the trial results.^16^, ^17^

From an ethical standpoint, the three DBPCTs were performed in accordance with good clinical practice defined by the International Council for Harmonization and the principles that have their origin in the Declaration of Helsinki and local laws and regulations. All participants or parents or legal representatives (for participants 17 years or younger) gave their written consent to participation after being informed of the trial objectives and procedures.

### 2.2 Study endpoints and new assessment score

The EAACI‐recommended CSMS^15^ reflects the symptom severity as well as the intake of rescue medication considering a stepwise‐simplified approach based on the clinical effects of pharmacotherapy on symptom reduction. The recommended scoring system for the CSMS is based on an equal weight of the total daily symptom score (dSS) and the total daily medication score (dMS). The dSS uses a well‐defined and easy‐to‐understand terminology for nasal symptoms (itchy nose, sneezing, runny nose, blocked nose) and conjunctival symptoms (itchy/red eyes, watery eyes). Each symptom score ranges on a 0–3 scale as follows: 0 = no symptoms; 1 = mild symptoms (sign/symptom clearly present, but minimal awareness; easily tolerated); 2 = moderate symptoms (definite awareness of sign/symptom that is bothersome but tolerable); 3 = severe symptoms (sign/symptom that is hard to tolerate; causes interference with activities of daily living and/or sleeping). The maximum score for the total dSS (sum of the individual symptom scores) is 3 (i.e. 18 points/divided by 6 symptoms) = dSS_0‐3_.^15^ A stepwise use of rescue medication is summed up in the dMS based on the Allergic Rhinitis and its Impact on Asthma in collaboration with the World Allergy Organization (WAO) recommendations in giving rescue medication.^4^, ^18^ Accordingly, the score assigned for each medication is 1 for oral and/or topical (eyes or nose) non‐sedative H1 antihistamines (H1A); 2 for intra‐nasal corticosteroids (INCS) with/without H1A; 3 for oral corticosteroids with/without INCS, with/without H1A. The total dMS ranges from 0 to 3 (maximum score) = dMS_0‐3_.

Consequently, the total daily CSMS ranges on a 0–6 scale: CSMS_0‐6_ = dSS_0‐3_ + dMS_0‐3_.

The 3 DBPCTs captured all the necessary information so that it was possible to assess the efficacy of the 5‐grass tablet in this post hoc analysis using the recommended CSMS. Based on the daily diary cards completed by the patients, the rhinoconjunctivitis total symptom score (RTSS_0‐18_) equivalent to the dSS without dividing by 6, and the rescue medication score (RMS_0‐3_) equivalent to the dMS with the same stepwise approach were calculated over the pollen period. The average RTSS_0‐18_ over the pollen period was the primary endpoint for CT1 and CT3.^12^, ^14^ Another symptom score was also analyzed in the DBPCTs, the adjusted symptom score (AdSS_0‐18_), which adjusted the RTSS for rescue medication use). Briefly, the AdSS_0‐18_ took into account the highest RTSS score on the preceding day and applied it to the day on which the rescue medication was taken and the following day.^19^ The average AdSS_0‐18_ over the Year 3 pollen period was the primary endpoint in CT2.^13^ For analyzing the proposed CSMS in the 3 DBPCTs, we calculated the balanced score on a scale from 0 to 36 as follows: daily CSMS_0‐36_ = [daily RTSS_0‐18_ + (daily RMS_0‐3_)*6].

### 2.3 Statistical model

As per the primary endpoints of the respective studies, an analysis of covariance (ANCOVA) was used to statistically assess the CSMS_0‐36_ over the primary period (i.e. the pollen period while on treatment in CT1 and CT3 and the Year 3 pollen period in CT2) in a modified Intention‐to‐Treat (ITT), defined as all patients who received at least one dose of the investigational product and had recorded the primary efficacy measure on at least one day during the primary period. The ANCOVA model used treatment as the main effect, pooled study centre as the stratification factor and age, gender, sensitization, and asthma status as covariates. A point estimate and 95% confidence interval (CI) for the difference in the adjusted least square (LS) means between the active treatment and placebo groups were calculated. The relative LS mean difference (%) was calculated as follows: 100 × (LS mean 300 IR—LS mean placebo)/LS mean placebo. For the analyses, the probability of type I error (*α*) was set at 0.05. All inferential tests were two‐sided. Statistical analysis was performed using SAS software, version 9.4 (SAS Institute, Inc).

### 2.4 Clinical relevance

The clinical relevance of the treatment effect (i.e. the reduction in symptom and medication score with the 300 IR 5‐grass tablet vs. placebo over the primary period) can be defined as the smallest reduction in the combined score likely to be important from the patients' perspective. The probability of the reduction in the score actually observed in the 3 CTs was calculated from the reduced centred normal distribution of the LS mean differences in average CSMS_0‐36_. Furthermore, the observed reduction in CSMS_0‐36_ in the 3 CTs was translated into a clinically relevant improvement by considering either component of the combined score: RTSS_0‐18_ or RMS_0‐18_. The reduction in RTSS_0‐18_ was correlated with a decrease in symptom severity, while the reduction in RMS_0‐18_ was correlated with a decrease in the number of days with less therapy for a patient taking antihistamines or nasal corticosteroids daily over the pollen period.

## 3 RESULTS

The analysis included 416 patients treated with the 300 IR 5‐grass tablet (136 in CT1, 149 in CT2, 131 in CT3) and 448 patients treated with placebo (148 in CT1, 165 in CT2, 135 in CT3) in the modified ITT.

### 3.1 Primary and main endpoints of the 3 DBPCTs

As previously published, the primary endpoints were the average RTSS_0‐18_ over the pollen period for CT1 and CT3 and the average AdSS_0‐18_ over the Year 3 pollen period for CT2.^12^, ^13^, ^14^ In the modified ITT, the treatment effect corresponding to the LS mean differences in the respective primary endpoints between the 300 IR 5‐grass tablet and placebo was statistically significant: average RTSS_0‐18_ point estimate −1.39 (95% CI [‐2.09; −0.69], *p* = 0.0001) in CT1, average AdSS_0‐18_ point estimate −1.81 (95% CI [−2.61; −1.02], *p* < 0.0001) in CT2, and average RTSS_0‐18_ point estimate −1.13 (95% CI [−1.80; −0.46], *p* = 0.001) in CT3. The relative LS mean differences versus placebo were −28.2%, −34.8%, and −25.5%, respectively. When analyzed as secondary endpoints according to the trials, the average RTSS_0‐18_ and AdSS_0‐18_ over the pollen period showed a similar magnitude of effect to that observed for the primary endpoint (Table 1). Regardless of the score, RTSS_0‐18_, AdSS_0‐18_ or RMS_0‐3_, positive results in favour of the 300 IR 5‐grass tablet were consistently noted across the studies.

**TABLE 1:** Symptom and medication scores (combined or not) over the pollen period in the 3 clinical trials (modified ITT).

	CT1	CT2	CT3
	300 IR *n* = 136	300 IR *n* = 149	300 IR *n* = 131
	Placebo *n* = 148	Placebo *n* = 165	Placebo *n* = 135
	Point estimate [95% CI], *p*‐value, relative LS mean difference	Point estimate [95% CI], *p*‐value, relative LS mean difference	Point estimate [95% CI], *p*‐value, relative LS mean difference
RTSS_0‐18_ ^a^	−1.39 [−2.09; −0.69]	−1.37 [−2.03; −0.71]	−1.13 [−1.80; −0.46]
	0.0001	<0.0001	0.0010
	−28.2%	−33.9%	−25.5%
RMS_0‐3_	−0.17 [−0.29; −0.05]	−0.16 [−0.25; −0.06]	−0.20 [−0.34; −0.06]
	0.0047	0.0011	0.0064
	−30.4%	−33.4%	−27.1%
AdSS_0‐18_ ^b^	−1.84 [−2.66; −1.02]	−1.81 [−2.61; −1.02]	−1.64 [−2.51; −0.78]
	<0.0001	<0.0001	0.0002
	−30.3%	−34.8%	−28.3%
CSMS_0‐36_	−2.51 [−3.88; −1.14]	−2.31 [−3.39; −1.23]	−2.31 [−3.58; −1.03]
	<0.0001	<0.0001	0.0004
	−29.7%	−33.8%	−26.3%

Abbreviations: AdSS_0‐18_, adjusted symptom score (scale 0–18); CSMS_0‐36_, combined symptom and medication score (scale 0–36); CT, clinical trial; IR, index of reactivity; LS, least square; mITT, modified intention‐to‐treat; *n*, number of patients in the mITT; RMS_0‐3_, rescue medication score (scale 0–3); RTSS_0‐18_, rhinoconjunctivitis total symptom score (scale 0–18).

^a^ The average RTSS_0‐18_ during the pollen period was the primary endpoint in CT1 and CT3.

^b^ The average AdSS_0‐18_ during the Year 3 pollen period was the primary endpoint in CT2.

### 3.2 Average CSMS_0‐36_ during the primary period

**FIGURE 1:**
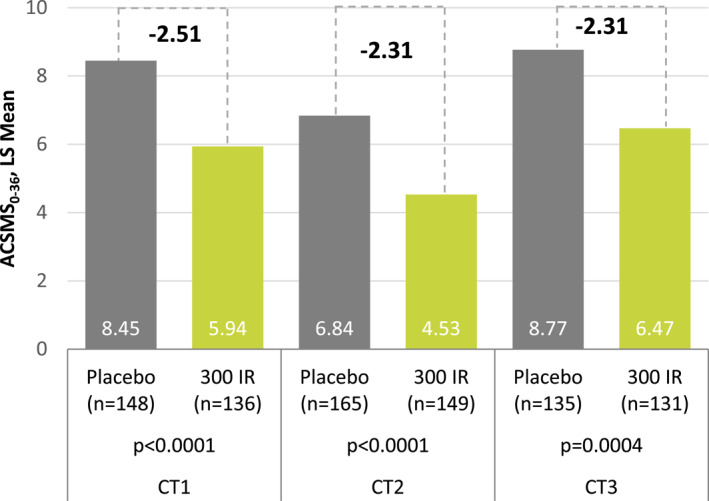
Average CSMS_0‐36_ (modified intention‐to‐treat (mITT)).

Over the primary evaluation period of each trial, statistically significant absolute LS mean differences in the average CSMS_0‐36_ were observed between the 5‐grass tablet and placebo: point estimate −2.51 (95% CI [−3.88; −1.14], *p* < 0.0001) in CT1, −2.31 (95% CI [−3.39; −1.23], *p* < 0.0001) in CT2, and −2.31 (95% CI [−3.58; −1.03], *p* = 0.0004) in CT3 (Table 1 and Figure 1). The relative LS mean differences versus placebo were −29.7%, −33.8%, and −26.3%, respectively.

### 3.3 Clinical relevance

**FIGURE 2:**
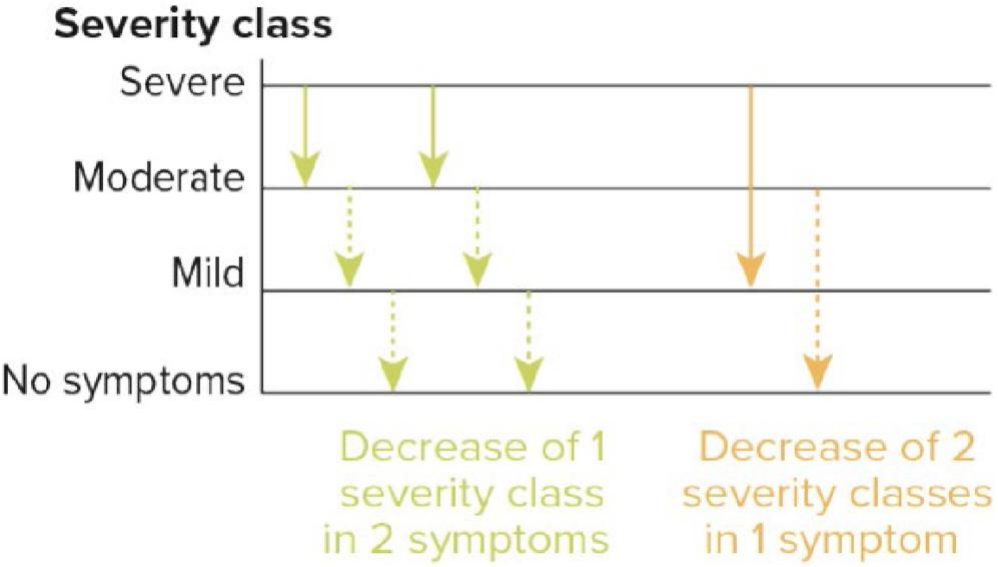
Schematic overview of the clinical relevance of the observed reduction in the average RTSS_0‐18_ (modified intention‐to‐treat (mITT)).

**FIGURE 3:**
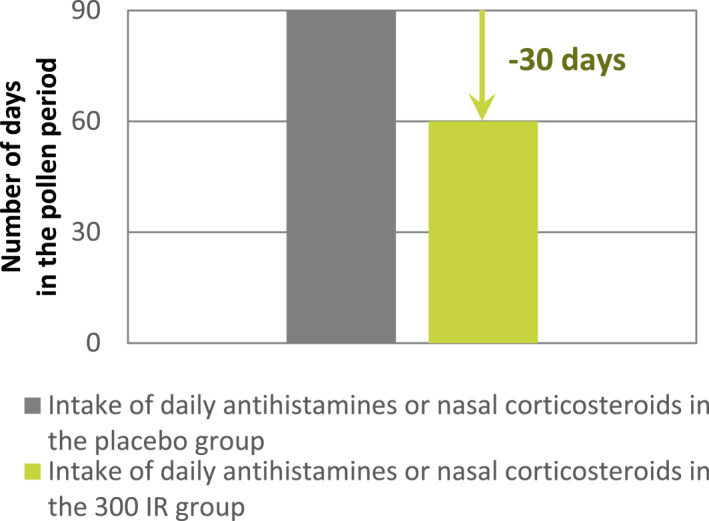
Schematic overview of the clinical relevance of the observed reduction in the average RMS_0‐18_ (modified intention‐to‐treat (mITT)).

In all 3 CTs, the probability of a reduction in the CSMS_0‐36_ of at least 2 points was 76.7%, 71.3%, and 68.2% as estimated from the reduced centred normal distribution of the LS mean differences in the average CSMS_0‐36_. This reduction was also translated into a clinically relevant improvement from the patients' perspective. Looking at the RTSS_0‐18_ component, a reduction of at least 2 points may reflect over the pollen period a decrease of 1 severity class (from severe to moderate, from moderate to mild or from mild to no symptoms) in 2 symptoms or a decrease of 2 severity classes (from severe to mild, from moderate to no symptoms) in 1 symptom, considering the other symptoms and rescue medication intake remain stable (Figure 2). Looking at the RMS_0‐18_ component, a reduction of at least 2 points may reflect around 10 days less therapy per month or 1 month less medication per 3 months over the pollen period for a patient taking antihistamines or nasal corticosteroids daily, considering that all symptoms remain stable (Figure 3).

## 4 DISCUSSION

Allergen immunotherapy reduces symptoms as well as the use of medication in the allergic individual.^2^, ^7^ In evaluating its efficacy in allergic diseases like grass pollen allergy in clinical trials, the recommended balanced combined score CSMS considers both the symptoms and medication intake. Because the use of rescue medication that is provided for ethical reasons has an impact on symptom severity/scores, it must be recorded daily and included in the scoring system according to the stepwise approach recommended by the WAO.^20^ In addition, the scores for symptoms and medication have to be balanced. All these requirements have been considered in the scoring system proposed by the EAACI task force.^15^ Using such a standardized scoring system gives the opportunity to directly compare different clinical trials.

The DBPCTs with the 300 IR 5‐grass tablet in children, adolescents and adults with grass pollen ARC with or without controlled asthma have demonstrated significant clinical efficacy, sustained efficacy and carry‐over effect using pre‐specified symptom scores as primary variables.^11^, ^12^, ^13^, ^14^ CT1 and CT3 were conducted following recommendations from the EMA ‘Guideline on the Clinical Development of Medicinal Products for the Treatment of Allergic Rhino‐conjunctivitis’ (EMA/CHMP/EWP/2455/02, 2004) as to use patient self‐rated symptom scores for primary efficacy measurement. Since then, the EMA ‘Guideline on the Clinical Development of Products for Specific Immunotherapy for the Treatment of Allergic Diseases’ was issued, recommending the use of a primary variable reflecting the treatment effect on both the symptoms and use of symptomatic medications (EMA/CHMP/EWP/18504/2006, 2008). While CT1 and CT3 were already completed, the long‐term CT2 was ongoing, and the study protocol was amended in the second year to opt for a primary endpoint reflecting both measures: the average AdSS_0‐18_.^19^ Though this score was accepted by European regulatory authorities, the comparison of treatment effect with other products in the field remained difficult since there was no clear guidance for combining symptoms and medication use leaving the room for using different methodologies.

Hence, the present post hoc analysis focussed on assessing the clinical efficacy of this grass SLIT tablet using the EAACI‐recommended combined score.^15^ Noteworthy, as the CSMS_0‐6_ was not yet universally used by all companies evaluating their product (different scales can be noted), it was assumed a 2‐point difference in score in the active group versus placebo that can be considered a relevant threshold for clinical relevance could be better perceived on a larger scale 0–36 (i.e. by multiplying the RMS by 6) rather than on a 0–6 scale (i.e. by dividing the RTSS by 6). The results showed a relative difference between 300 IR 5‐grass tablet and placebo in CSMS_0‐36_ over the primary period of −29.7%, −33.8%, and −26.3% in CT1, CT2, and CT3, respectively. Using this new scoring system, the observed magnitude of effect is consistent between the CTs as well as with that observed on their respective primary endpoints (−28.2%, −34.8%, and −25.5%).^12^, ^13^, ^14^ The treatment effect corresponding to the reduction in CSMS_0‐36_ between the 300 IR 5‐grass tablet and placebo is similar in all age groups (children, adolescents and adults) and in the European population compared to the overall population, as shown in CT2. Moreover, this positive effect appears to increase over time with 26%–30% observed on the first pollen period in CT1 and CT3 and 34% on the third pollen period in CT2.

Combined score results with another grass pollen SLIT tablet, the SQ‐standardized grass (*Phleum pratense*) allergy immunotherapy tablet (ALK‐Abelló, Hørsholm, Denmark), were reported from a randomized DBPC, multinational, phase III trial including adults (18–65 years old) with moderate‐severe grass pollen‐induced ARC.^21^ In this trial, patients received 3 years of continuous treatment, starting 4–8 months prior to the first pollen season. In this trial, a weighted rhinoconjunctivitis combined score (RCS) was calculated based on the 6 daily rhinoconjunctivitis symptom scores (total score ranging from 0 to 18) and the daily rhinoconjunctivitis symptomatic medication score (total score ranging from 0 to 36). The weighed RCS was reduced by −33% and −36% relative to placebo in the first and third grass pollen seasons, respectively.^21^ These results with a different combined score construct are consistent with those observed with the 300 IR 5‐grass tablet following 1 and 3 years of pre‐coseasonal treatment in adults (involving ca. 6 months treatment per year, rather than continuous treatment), starting ca. 4 months prior to the first pollen season. Noteworthy, when the total combined score was calculated by simply summing the rhinoconjunctivitis symptom and medication scores (=TCS_0‐54_), a similar relative reduction versus placebo was observed (−34% on average).^22^ In a trial comparing the 1‐grass tablet to placebo in children aged 5–16 years with grass pollen‐induced ARC, a relative difference from placebo of −24.2% was observed in the TCS_0‐54_ (post hoc analysis),^22^ which is also consistent with the results obtained in the paediatric trial with the 5‐grass pollen tablet.

Another important question for the treatment of allergic patients is whether a significant effect over a placebo is also clinically meaningful from the patient's perspective that is, a definition of responders to treatment.^23^ In other words, to what extent can the treatment effect be perceived by the patient in relation to the severity of bothersome symptoms and/or the use of rescue medications that may have adverse effects. Firstly, it is important to note that the estimated proportion of patients treated with the 5‐grass pollen tablet and achieving a reduction in CSMS_0‐36_ of at least 2 points was substantial (more than 70% in adults, ∼68% in paediatric patients), supporting the relevance of this threshold for clinical interpretation. This is made possible considering the two components of the CSMS_0‐36_. The reduction of at least 2 points in the RTSS_0‐18_ may reflect a decrease of 1 severity class in 2 symptoms or a decrease of 2 severity classes in 1 symptom over the pollen period. Alternately, the reduction of at least 2 points in the RMS_0‐18_ may reflect around 1 month less medication per 3‐month pollen season for a patient taking antihistamines or nasal corticosteroids daily. Such an improvement in symptoms and decrease in medication use is meaningful for the patient and thus can be regarded as clinically relevant. This could be seen with the 300 IR 5‐grass pollen tablet in all age groups. It is important to remind that beyond a clinical interpretation based on absolute score differences between the active treatment and placebo, knowledge of patients' perceptions remains essential to accurately assess the treatment benefit. In the 3 reported CTs, the improvement in quality of life and/or the global evaluation of the treatment effect assessed by the patients were consistently in favour of the 300 IR 5‐grass SLIT tablet.^12^, ^13^, ^24^, ^25^ Moreover, in a real‐word evidence study in grass pollen ARC patients, the vast majority of adults, adolescents and children treated with the SLIT tablet achieved a relevant benefit as indicated by a Patient Benefit Index score measuring patients' expectations and satisfaction through specific questionnaires.^26^ Altogether, these data established from patients' perspectives support the clinical relevance of the 300 IR 5‐grass SLIT tablet.

The clinical relevance of the 300 IR 5‐grass tablet is also reinforced by the fact that the magnitude of the effect is consistent, irrespective of the score assessed, maintained, or even increased over the treatment course. Indeed, such a consolidation of the effect over 3 years of therapy is key to ensure long‐lasting meaningful benefits even after treatment cessation,^27^ in line with EAACI guidelines which recommend a minimum of 3 years of AIT to achieve long‐term efficacy.^2^ Considering that patients' willingness to commit and persist with several years of AIT remains a challenge, strengthening their education and participation (shared decision making) is crucial to help attain this goal by covering the patients' specific needs and preferences.^1^, ^27^, ^28^ Noteworthy, the 5‐grass SLIT tablet offers the advantage of a discontinuous pre‐coseasonal regimen over perennial treatments, which may improve patient adherence.^29^

The strength of the present analysis is that it was performed in line with the recommendations of Health Authorities and international experts to combine a symptom scoring together with a medication scoring in an equal manner for the analysis of the primary endpoint in field trials.^20^, ^29^ Though the DBPCTs did not include such a balanced combined score as primary endpoint, it was possible to calculate it post hoc based on the reported rhinoconjunctivitis symptoms and medication scores. Indeed, the symptom and medication scoring systems predefined in the current trials follow those endorsed by the FDA and EMA and used to establish the EAACI Task Force‐recommended CSMS. Notably, the medication scale was chosen according to a stepwise approach and was comparable with that used in other trials with SLIT products.^30^, ^31^, ^32^, ^33^ The results of this analysis consistently confirm the main outcomes of the trials as published previously.

The EAACI‐standardized combined score remains to be further validated in more studies in the field, notably in paediatric trials.^5^ However, its use allowed direct comparison between CTs and enabled an interpretation of the clinical relevance of the treatment effect of the 300 IR 5‐grass tablet in line with the current state of the art. Further analyses using recommended methodologies are needed to warrant the proposed threshold for the CSMS_0‐36_ as minimal clinically important difference.^34^, ^35^

## 5 CONCLUSION

The post hoc analyses in 3 randomized DBPCTs with the 300 IR 5‐grass pollen SLIT tablet showed a reduction of above 2 points in the average CSMS_0‐36_ over the primary period compared to placebo in patients from 5 years of age with grass pollen‐induced ARC with or without controlled asthma. The results were not only statistically significant but can be considered clinically meaningful from the patient's perspective. The clinical relevance of the 300 IR 5‐grass tablet is thus reinforced, enabling allergy specialists to offer a valuable option of treatment for grass pollen allergy tailored to the patients' needs.

## AUTHOR CONTRIBUTIONS

All authors have made substantial contributions to the analysis or interpretation of data, critically reviewed the manuscript for important intellectual content and approved the final version for publication. These authors had full access to all the data in the study and take responsibility for the integrity of the data and the accuracy of the data analysis.

## CONFLICT OF INTEREST STATEMENT

**O Pfaar** reports grants and/or personal fees from ALK‐Abelló, Allergopharma, Stallergenes Greer, HAL Allergy Holding B.V./HAL Allergie GmbH, Bencard Allergie GmbH/Allergy Therapeutics, Lofarma, ASIT Biotech Tools S.A., Laboratorios LETI/LETI Pharma, GlaxoSmithKline, ROXALL Medizin, Novartis, Sanofi‐Aventis und Sanofi‐Genzyme, Med Update Europe GmbH, streamedup! GmbH, Pohl‐Boskamp, Inmunotek S.L., John Wiley and Sons, AS, Paul‐Martini‐Stiftung (PMS), Regeneron Pharmaceuticals Inc., RG Aerztefortbildung, Institut für Disease Management, Springer GmbH, AstraZeneca, IQVIA Commercial, Ingress Health, Wort&Bild Verlag, Verlag ME, Procter&Gamble, ALTAMIRA, Meinhardt Congress GmbH, Deutsche Forschungsgemeinschaft, Thieme, Deutsche AllergieLiga e.V., AeDA, Alfried‐Krupp Krankenhaus, Red Maple Trials Inc., Königlich Dänisches Generalkonsulat, Medizinische Hochschule Hannover, ECM Expro&Conference Management, Technische Universität Dresden, Lilly, Paul Ehrlich Institut, all outside the submitted work and within the last 36 months; and he is member of EAACI Excom, member of ext. Board of directors DGAKI; coordinator, main‐ or co‐author of different position papers and guidelines in rhinology, allergology and allergen‐immunotherapy. **U. Wahn** reports personal fees from ALK‐Abelló, personal fees from Berlin‐Chemie, Novartis, Stallergenes Greer and Viatris, outside the submitted work. **G.W. Canonica** has nothing to disclose for this paper. **F. Bahbah** is a former employee of Stallergenes Greer and reports personal fees and non‐financial support from Stallergenes Greer during her time with the company. **P. Devillier** reports personal fees and non‐financial supports from ALK‐ Abelló, Astra Zeneca, Chiesi, GlaxoSmithKline, IQVIA, Menarini, Procter & Gamble, Stallergenes Greer and Viatris.

## Data Availability

The data that support the findings of this study are available from the corresponding author upon reasonable request.
